# In Search of Basement Indicators from Street View Imagery Data: An Investigation of Data Sources and Analysis Strategies

**DOI:** 10.1007/s13218-022-00792-4

**Published:** 2023-01-20

**Authors:** Anh Vu Vo, Michela Bertolotto, Ulrich Ofterdinger, Debra F. Laefer

**Affiliations:** 1 School of Computer Science, University College Dublin, Belfield, Dublin 4, D04 V1W8 Dublin, Ireland; 2 School of Natural and Built Environment, Queen’s University Belfast, Stranmillis Road, Belfast, BT 95AG Northern Ireland; 3 Center for Urban Science & Progress, New York University, 370 Jay Street, Brooklyn, NY 11201 USA; 4 Department of Civil and Urban Engineering, New York University, 6 MetroTech Center, Brooklyn, NY 11201 USA

**Keywords:** Street-view, Imagery, Flood, Image segmentation, Scene reconstruction, Data quality

## Abstract

Street view imagery databases such as Google Street View, Mapillary, and Karta View provide great spatial and temporal coverage for many cities globally. Those data, when coupled with appropriate computer vision algorithms, can provide an effective means to analyse aspects of the urban environment at scale. As an effort to enhance current practices in urban flood risk assessment, this project investigates a potential use of street view imagery data to identify building features that indicate buildings’ vulnerability to flooding (e.g., basements and semi-basements). In particular, this paper discusses (1) building features indicating the presence of basement structures, (2) available imagery data sources capturing those features, and (3) computer vision algorithms capable of automatically detecting the features of interest. The paper also reviews existing methods for reconstructing geometry representations of the extracted features from images and potential approaches to account for data quality issues. Preliminary experiments were conducted, which confirmed the usability of the freely available Mapillary images for detecting basement railings as an example type of basement features, as well as geolocating the features.

## Introduction

Of all natural disasters, flooding has the greatest potential of causing damage and affecting the greatest number of people worldwide [1]. Flooding cannot be completely prevented but its consequences can be mitigated. Recent advances in computer science, and particularly in artificial intelligence (AI) are being employed to improve flood risk management and preparedness. For example, Lopez-Fuentes et al. [2] and Ergani et al. [3] introduced deep learning approaches to detect floods and segment water bodies from social media images to aid flood emergency handling. Another example is the application of Convolutional Neural Network (CNN) on a fusion of digital elevation models, optical, and radar satellite images to map the spatial extents of floodings at a large scale [4].

Assessing flood risks is an important step in its management. Assessment requires knowledge of the sources and flow paths of floodwaters, as well as the nature of receptors that may be impacted (e.g., residents, infrastructure assets and others). This project aims to explore AI approaches and large-scale, street-view imagery (SVI) datasets (i.e., images captured from the perspective of pedestrians or road vehicles) to enhance flood risk assessment. In particular, the focus is on automatic identification of building basement structures from SVIs. While basements affect ground water and surface water flows, most flood assessment models omit them. Furthermore, basement inhabitants and properties are at a higher risk of flooding [5]. Basements are inundated early in a flooding event and can fill quickly due to their limited volumes. Thus, information of basement spaces extracted in this project can improve flood risk assessments for individual property owners and their surrounding communities.

**Fig. 1:**
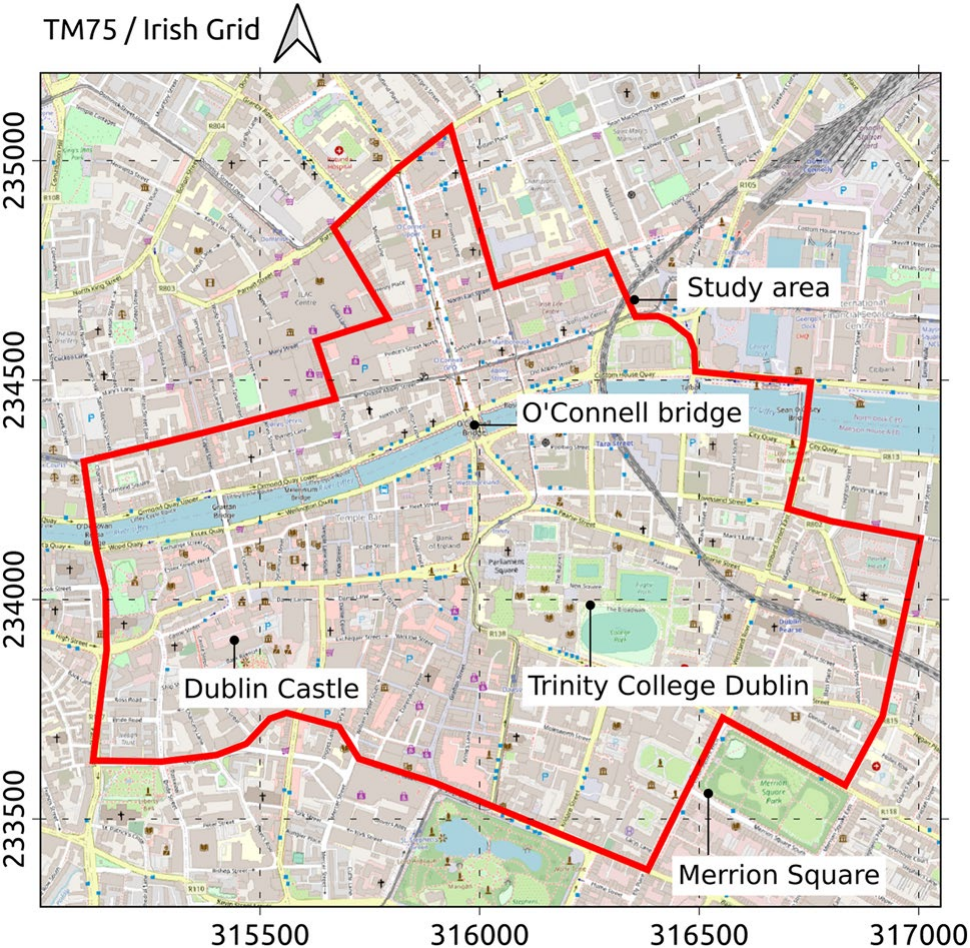
Study area in Dublin city, Ireland. Map data from OpenStreetMap

Since multiple SVI datasets such as Google Street View, Mapillary, and Karta View are available for most towns and cities globally, this paper’s proposed methodology is designed to be readily transferable, wherever suitable data are available. However, every data source has its own limitations. Section 2 of this paper provides a critical review of the SVI data sources with respect to their suitability for the project’s purpose. An area of approximately 2 km$^{2}$ in the Dublin city centre in Ireland (Fig. 1) is selected as a study area for this project. Basement flooding is a known concern there. The 2005 Greater Dublin Strategic Drainage Study highlighted a number of issues with regard to basements and their flooding risks. Basement flooding risk was also identified in the Strategic Flood Risk Assessment (SFRA) conducted for the Dublin City Development Plan 2022–2028 [6]. The area has a long history of past flood events^1^. For instance, during a single river overtopping incident on 24/10/2011, 55 basements were reported to be flooded^2^.

Both the current absence of a database of basements for the Dublin region and the criticality of having such information are confirmed in Sections 2.3 and 2.7 in Volume 6 of the 2005 Greater Dublin Strategic Drainage Study report [7]. The most important information necessary for flood risk management includes the location, level, and use of basements. Dublin City Council has limited information of approximately 16,200 basement structures in the area between the Grand Canal and Royal Canal in the city centre. The report recognised that the actual number of basements in Dublin is far beyond what is currently captured. Basement information in South Dublin, Fingal, Dun Laoghaire-Rathdown and Bray counties is absent. An AI model learning to detect basement structures from SVI data would be very useful in this context. Such a model can learn the visual representation of basements in areas where basement information is available and apply the model to predict the presence of basements in areas where the information is absent. This is especially feasible when the difference in the architectural styles is not significant so that the data available for training can sufficiently represent the target data.

The goal of the project is to exploit large scale SVI data sources and the latest computer vision algorithms to enrich information of basement structures and enhance knowledge of flood risk. To achieve that goal, the project seeks to pursue the three questions:

- What features visible from the street level can indicate the presence of a basement in a building?
- Which SVI data sources are available and suitable for indicating the features?
- Which computer vision algorithms are capable of automatically detecting the features of interest?

The project is motivated by the underuse of imagery data for the detection of basement structures. Searching the Scopus database in November 2022 for research of all types matching three terms “basement”, “detection”, and “image” resulted in 196 items. However, none of the resulting items was relevant to the topic in question. While there were multiple studies related to detecting and extracting basement information, all employed 3D laser scanning data and/or ground-penetrating radar (GPR) data (e.g., [8–10]) which are more expensive and not as widely available as imagery data.

## Presence of Basements as a Flood Risk Indicator

Flood risk is a function of hazard, exposure, and vulnerability [11]. The presence of a basement structure in a building does not automatically mean that the building is at a risk of flooding. Other factors such as the probability of flooding in the area where the building locates, basement configuration, use, and surrounding built and natural features are critical in determining actual flood risk. Nevertheless, the presence of a basement is a factor in contributing to flood risk of a building [12–14]. Multiple existing flood assessment models account for the presence of basement structures. Examples include the HAZUS model by the US Federal Emergency Management Agency [15], the Multi-Coloured Manual by Penning-Rowsell et al. [16], and the US Army Corps of Engineers’ model [17]. While being necessary for flood risk assessment, basement information is not available in many places. The research presented in this paper aims to fill the gap by extracting basement information for flood assessment models such as those aforementioned.

Apart from being at risk of flooding themselves, basement structures may also impact on the groundwater and surface water flows during a flood event [18]. A basement also affects the structural stability of buildings in its surrounding. In Dublin, the development of basements is disallowed in areas where the probability of flooding is higher than 0.1% for river flooding or 0.5% for coastal flooding [6]. A basement impact assessment is compulsory for any building development proposal that includes a basement. The priority risk posed by basements has been acknowledged as part of flood evacuation models (e.g., [19–21]). However, the presence of basement structures has commonly not been implicitly accounted for in numerical flood prediction models.

**Fig. 2:**
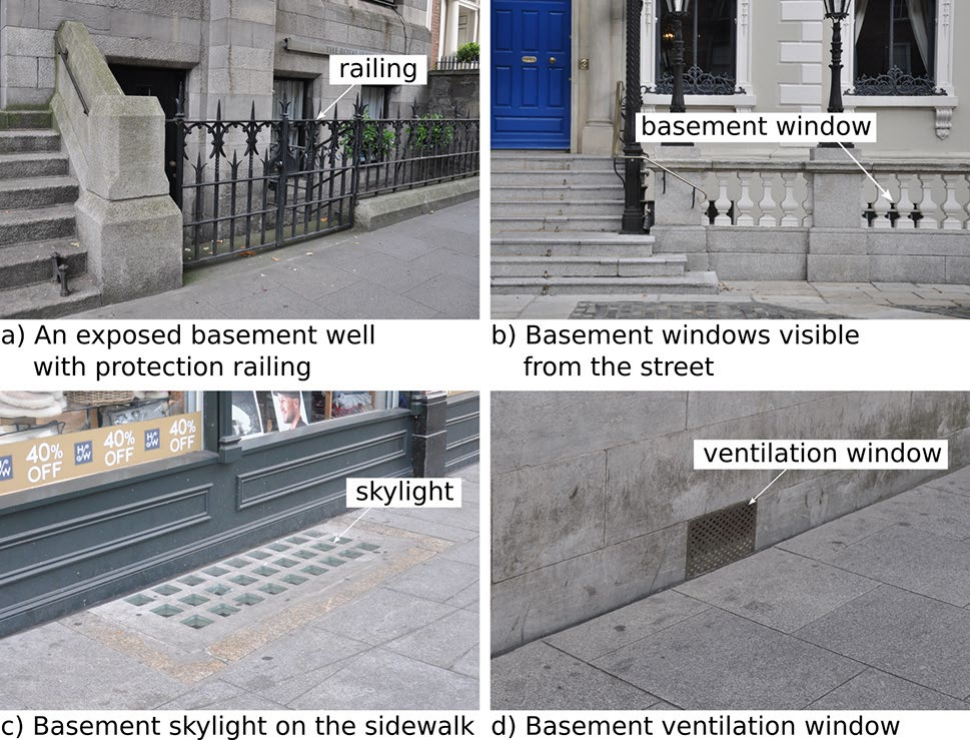
Visual features indicating potential presence of a basement

The presence of a basement in some buildings is visible from the street view. In some cases, the basements themselves can be directly seen from the street. Figure 2a shows an example of such a case, in which the Georgian terrace houses have exposed basement structures identifiable through the basement wells, basement windows, and protection railings. When not fully exposed, some basements can be identified through features such as ventilation shafts, airbricks on the façades and skylights on the sidewalk. Those features are common and essential in providing air and natural lighting from the outside space to the subterranean spaces. The aforementioned visual features are present in many basements while not every basement structure has one of the features. This project will focus on identifying basements through their visual features exposed to the street view. However, even when no visual features exist on the exterior of a building, the existence of a basement can potentially be predicted from the architecture style, and other information such as construction time and information of nearby buildings. Previous works such as [22] has proved that some information about a scene can be inferred from an image of the scene without explicit visibility of relevant objects in the image.

## Street-View Imagery Databases

Street view imagery data are available in many towns and cities around the world [23]. SVI images are captured from the street level and from much closer to the objects of interest (i.e., building façades) compared to satellite and aerial images. Due to the favourable point of view and the high availability, SVI data allow capturing many features of interest described in Section 2. SVI data have been successfully used in previous works to analyse different aspects of the built environment such as architectural style, building age, and building energy efficiency [24, 25].

There are a number of SVI data providers. Major providers include Google Street View, Apple Look Around, Mapillary, and KartaView which provide coverages in multiple countries. There are other providers offering data at a smaller scale such as CycloMedia which provides panoramic SVI data for most of the Netherlands and several cities in Europe and the United States, Tencent Maps and Baidu Maps offering data mostly for China, and Kakao Maps offering SVI data in Korea. Based on primary screening, the data providers potentially suitable for this project are Google Street View and Mapillary. Apple Look Around does not provide an Application Programming Interface (API) for data download. CycloMedia data are not available for the area of interest (AOI) in Dublin and KartaView data are very sparse in the AOI.

The remainder of this section discusses the two top candidates, GSV and Mapillary, in detail. In particular, GSV and Mapillary data and services are compared with respect to a set of criteria deemed important to the project. The seven recommended criteria for pre-screening the data sources are as follows. The word “must” indicates criteria deemed mandatory while “should” indicates criteria that are desirable but not mandatory.

- Criterion 1—The data should be downloadable in batch such as via an API.
- Criterion 2—Necessary metadata must be available and contain sufficient information to identify and georeference objects in the images.
- Criterion 3—The spatial coverage must include the study area.
- Criterion 4—The temporal coverage should be as extensive as possible to reflect the changes in the built infrastructure configuration.
- Criterion 5—The image resolution and quality must be sufficient to allow extracting the necessary basement information.
- Criterion 6—Permission must be obtainable from the data providers to use the data in this project.
- Criterion 7—Data acquisition costs should not be prohibitive.

### Overview of GSV and Mapillary

GSV is the most popular SVI data service. According to a survey by Biljecki and Ito [26], GSI is the dominant source of SVI data among urban studies research. The use of GSV images has been seen in hundreds of academic papers.

While also offering geo-tagged, street-level imagery similar to GSV, Mapillary sources data from volunteer contributors. Due to the data’s origin, Mapillary images are very diverse in terms of capturing equipment (e.g., mobile phone cameras, dashcams, action cameras), moving platforms (e.g., cars, buses, bicycles, pedestrians), as well as image quality.

### APIs and Data Offered Through APIs

Both GSV and Mapillary provide APIs for downloading images as well as image metadata. Both services satisfy Criterion 1 regarding data download-ability. The majority of GSV data are panoramic while most Mapillary images are flat. The Google’s Street View Static API supports queries by either location or street address. The capability to support queries by street address is especially useful in this project given the objects of interest are buildings and their features. Valuable information in the metadata response include the capture date and the geographic coordinates of the platform at the time the image was captured. The largest image size available through the Google Street View Static API is 640 $\times$ 640 pixels. Despite the image size restriction, retrieving a higher resolution view of smaller parts of an image is possible by reducing the FOV.

Mapillary provides an API as well as a Python Software Development Kit (SDK) to interact with its data. The current Mapillary API version 4 only supports window queries for image search. With the Python SDK, we can also search for images within a non-rectilinear AOI, search for images near a given location, and search for images looking at a given location. The last type of query is especially useful when we need to retrieve images of a building. While Mapillary does not support search by street address like GSV, the available search functionalities together with a third-party geo-coding service should allow search by address.

Mapillary images can be downloaded at the original sizes or normalised sizes (e.g., 256, 1024, 2048 pixels). The original image sizes are highly diverse as they are dependent on the capturing equipment. Image metadata offered by Mapillary are very comprehensive and consist of more than 23 metadata fields, including spatial information (coordinates, altitude, compass angle, orientation), temporal information (captured time), camera type and camera parameters. In addition to the spatial information originally recorded in the uploads, Mapillary performs its internal processing of the information and makes available the corrected values (e.g., corrected geometry, corrected altitude, corrected compass angle, etc.). Notably, the position and orientation information provided by Mapillary is of the cameras and not of the objects appearing in the images.

Mapillary also processes images uploaded on its platform using semantic segmentation, object detection as well as Structure from Motion (SfM) [27] to reconstruct 3D representations of the captured scenes. Some of the derived information, such as the SfM point cloud and entities detected by Mapillary object detection, can be accessed through the image metadata. Among over 60 classes of objects offered by Mapillary, building, wall, fence, guard rail, road, road curb, and sidewalk are potentially useful for our project. Objects of those classes when detected can provide a context to understand the image and can potentially be incorporated into the detection of the features of interest in our project. In summary, with respect to Criterion 2 (Metadata), Mapillary is more comprehensive even though the georeference information can be less accurate.

### Spatial and Temporal Coverage

The APIs described in Section 3 allow checking the data metadata for the coverage of each service. As of the time of writing, there were 6,800 GSV panoramic images and 17,613 Mapillary images within the area.

**Fig. 3:**
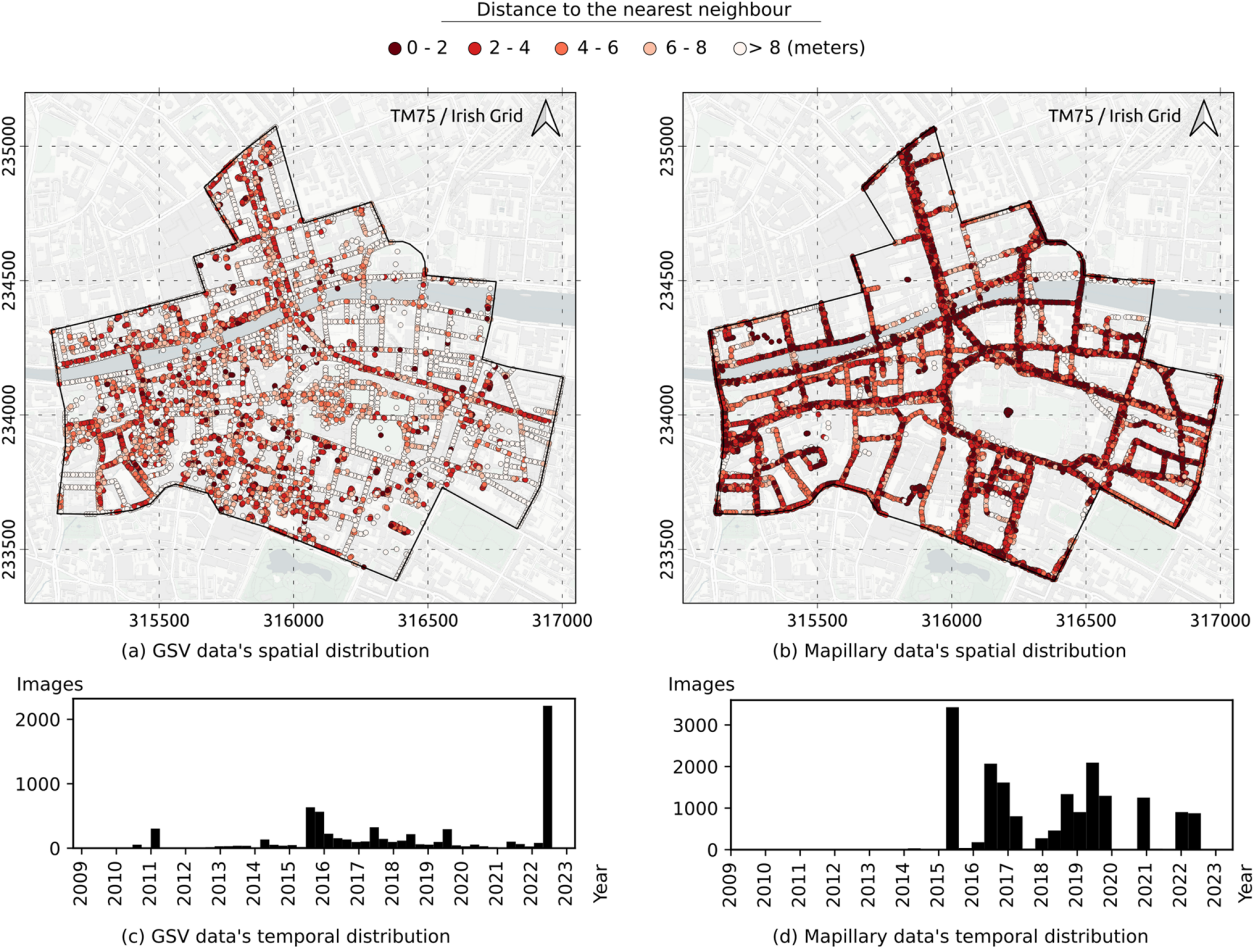
Spatial and temporal distributions of GSV and Mapillary data in the area of interest. Map data from Carto

The locations of GSV and Mapillary images are plotted in Fig. 3a and b. In general, both datasets cover most major roads in the AOI (i.e., satisfying Criterion 3 - Spatial coverage). GSV better covers smaller roads thereby capturing more buildings. The colours in the figures represent the distance of each data point to its nearest neighbour. The distance from a point to its nearest neighbour is inversely proportional to the local data density at that point. Darker colours in the images represent higher density values and vice versa. Mapillary image data points are more closely spaced. The median distance to the nearest neighbour is 1.8 m for the Mapillary data and 6.8 m for the GSV data. The temporal distribution of the two datasets are shown in Fig. 3c and d. A significant proportion of GSV images currently accessible through the API (i.e., 34.5%) are less than one year old (i.e., captured on or after 7/9/2022). The first significant batch of Mapillary uploads in the AOI occurred in 2015. Since then, new images have been contributed almost continuously to the platform except for the period from early 2020 to mid-2021. In general, the temporal distribution of the Mapillary dataset is more even compared to the GSV dataset i.e., better satisfies Criterion 4—Temporal coverage.

### Image Quality and Field of View

To be useful for the purpose intended in the project, an image should capture enough information of the objects of interest (i.e., buildings and their features) without being obstructed. The captured information should not be affected too much by issues such as blurriness, noise, inappropriate contrast, colour inaccuracy, distorsion, and flare. Being acquired by professional camera teams using specialised equipment, GSV images usually have sufficient quality (i.e., satisfying Criterion 5). In addition, the panoramic support allows setting and tuning the FOV post acquisition to achieve a favourable view of a building or specific building features.

**Fig. 4:**
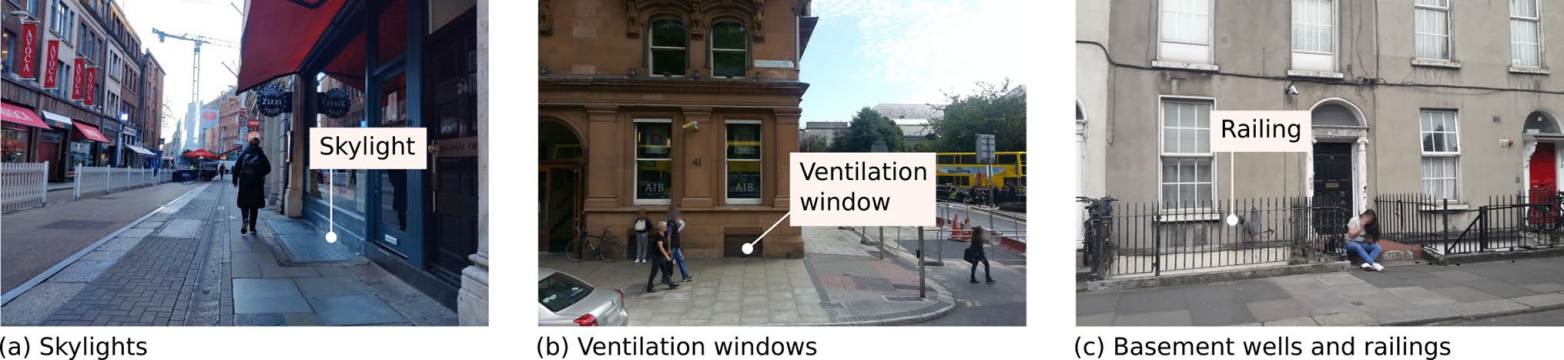
Examples of mapillary images with suitable quality and FOV for capturing basement features

**Fig. 5:**
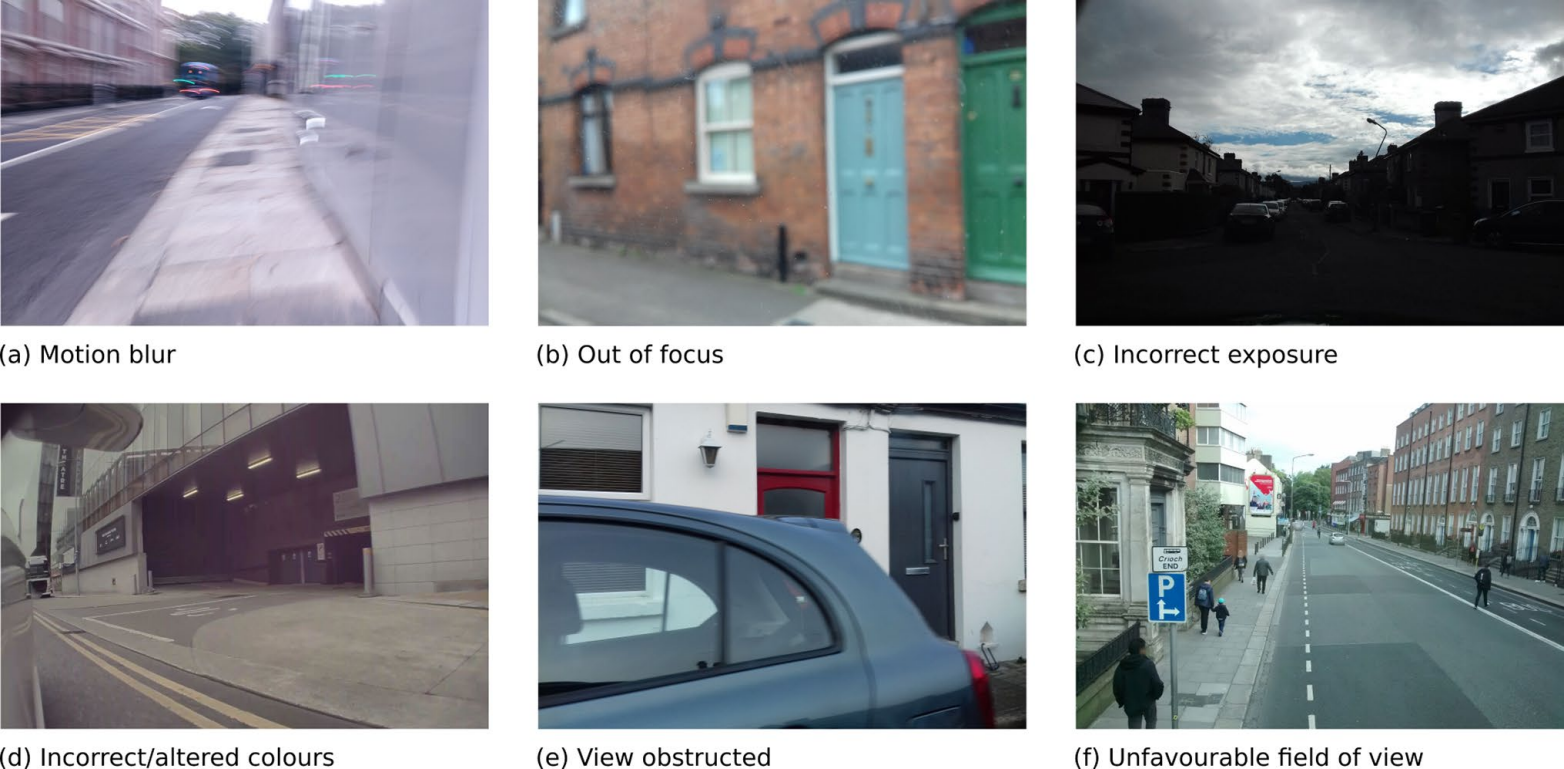
Example of mapillary images that suffer from quality issues

In contrast, Mapillary images are contributed primarily by amateurs using a wide variety of equipment and platforms. Similar to other sources of Volunteered Geographic Information (e.g., Open Street Map), Mapillary data do not come with a high level of consistency in data quality. The data quality of Mapillary imagery is highly diverse. There are high quality images suitable for the intended purpose such as those shown in Fig. 4. The technical quality and the FOV allow capturing basement identification features such as basement skylights, ventilation windows, basement wells and iron railings. However many Mapillary images suffer from various data quality issues such as motion blur (e.g., Fig. 5a), incorrect focus (e.g., Fig. 5b), incorrect camera exposure settings leading to images too bright or too dark (e.g., Fig. 5c), and incorrect colour settings or image colours altered post capture (e.g., Fig. 5d).

There are also other issues that hinder the usefulness of Mapillary imagery data in this project. For example, the object of interest (i.e., the building façade) in Fig. 5e was obstructed. In Fig. 5f, while the basement wells and raillings were captured, they occupy only small parts of the image. The latter issue is common as many Mapillary images were captured with the cameras aiming in the street direction. Ultimately, while working with the Mapillary dataset of the AOI, we encountered a considerable number of corrupted image files. There are also duplicate image files and nearly identical images (e.g., consecutive images captured while the camera is stationary) in the database. While those various issues do not completely impede the use of Mapillary data in the project, the issues should be considered/addressed in the data analysis strategy.

### Data Usage Permissions

Mapillary data are free of charge and are licensed under the Creative Commons Attribution-ShareAlike 4.0 International Licence (CC BY-SA 4.0). The highly permissive license allows copy, redistribute and build upon the licensed data for any purpose including commercial purposes. The licence gives all the permissions necessary for the purpose intended in the project (i.e., satisfying Criteria 6 &7). The licence requires proper attribution to the data source, indication of any changes made to the data, and distribution of derived data under the same licence.

Unlike Mapillary data, GSV data are commercial. At the time of writing, the cost for requesting a set of 1000 panoramic images through the GSV Static API is 5.6–7.0 US dollars, depending on the number of requests calculated monthly. Requests of metadata only are free of charge. Importantly, the Terms of Service do not allow deriving new information from GSV data or making offline copy of the data^3^. The restrictions apply to academic as well as commercial uses of the service. Deriving basement information from GSV imagery, therefore, is not an option in the project (i.e., mandatory Criterion 7 cannot be met). Nevertheless, we find the discussion of GSV data valuable for the following reasons. Firstly, GSV data represent an almost ideal street view data source. Analysing the characteristics of GSV data helps identify issues in other street-view data sources and identify possible solutions to address the issues. Secondly, while GSV’s Terms of Service do not allow downloading the images, the images accessible online are still very valuable as a reference for annotating other data sources. Thirdly, the restrictions in GSV’s Terms of Service are misinterpreted by many researchers. The discussion on the restrictions can be helpful to people who consider GSV data in future projects. That also highlights the barrier posed by proprietary datasets and emphasises the importance of open data.

## Developing a Data Analysis Strategy

As discussed in Section 3, GSV cannot be used as an imagery data source for extracting basement features due to the Terms of Service. Mapillary is the only possible dataset. Comparing the accuracy achievable with data from different SVI services is not possible. There are 17,613 Mapillary images available in the AOI at the time of writing. The data quality is highly heterogeneous with issues ranging from inappropriate camera settings, unfavourable FOV, to data corruption and duplication. Nevertheless, many Mapillary images have sufficient quality and provide valuable information of building basement features. This section discusses the development of a data analysis strategy to use Mapillary data as a means to achieve the project aim stated in Sections 1, 2 with considerations of the data quality issues mentioned in Section 3.

### Formulating the Target Tasks

The image analysis needs to identify basement features from an input geo-tagged image (i.e., Task 1) and reconstruct the shape, size, and location of the identified features in the real world (i.e., Task 2). Since we do not only need to detect the presence of the objects of interest (i.e., basement features) and locate them in an image but also need to delineate the objects at the pixel level, the first task should be formulated as either an image segmentation problem as opposed to classification, or object detection. Either semantic segmentation, instance segmentation, or panoptic segmentation could be sufficient for the project’s purpose. The second task is known as image-based scene reconstruction, or sometimes depth reconstruction or 3D reconstruction. Scene reconstruction allows consolidating information about any object detected in the first tasks. At the end of this task, basement information is available at the object level (e.g., building A has basement features visible in images 1, 2, 3) instead of at the image level at the end of Task 1 (e.g., images 1, 2, 3 contain features indicating a possible presence of a basement structure). Without performing the second task, one cannot attribute the detected features to specific buildings. Instance segmentation and scene reconstruction are common topics in computer vision.

### Task 1: Image Segmentation

There are numerous algorithms available for image segmentation. Traditional methods rely on hand-crafted features and rules such as thresholding [28], region growing [29], graph-cuts [30], and sparsity-based methods [31]. The advances in deep learning (DL) have created major breakthroughs in image segmentation. DL methods often well surpass traditional methods with respect to accuracy. Minaee et al. [32] and Ulku et al. [33] provided recent reviews of over 100 DL-based image segmentation methods. Such comprehensive reviews are valuable for selecting segmentation algorithms in this project. While a decision on which algorithm to use has not been reached at this stage, selection criteria have been discussed. What matters most to the task considered in the project is the segmentation accuracy, the capability to handle small object sizes, and the robustness against data heterogeneity and quality issues. Training and inference time requirements are not as critical because the project only deals with relatively static datasets and does not require real-time processing. In addition, the project favours DL methods that can exploit the global context information in an image (e.g., Pyramid Attention Network [34] and various extensions of YOLO (You Only Look Once) algorithm) over those that ignore the global context (e.g., conventional Fully Convolutional Networks [35]). The basement features (i.e., target objects) described in Section 2 are integral components within the surrounding environment including the buildings and the streetscape. The global context in an image is potentially useful in the project. Methods that are accompanied by well-maintained implementations, such as Mask RCNN [36], Panoptic FPN [37], Panoptic-DeepLab [38], YOLOv5^4^, and DetectoRS [39], may be given priority.

Given that Mapillary imagery data will be used, attention will be given to segmentation methods previously evaluated using Mapillary data. The Mapillary teams have carried out AI research and development. The company published the well-known Mapillary Vistas dataset for the first time in 2017. The current Mapillary Vistas version 2.0^5^ contained 25,000 SVIs annotated with 124 semantic object categories. The images are heterogeneous and represent different weather conditions, seasons, time, cameras and viewpoints. Together with the Cityscapes dataset [40], Mapillary Vistas is popular for benchmarking algorithms for segmenting images of urban scenes. Mapillary conducted and published its own research on image segmentation (e.g, [41]). The company has also co-organised multiple image segmentation competitions and workshops since 2017. Many teams participating in those competitions published their results in academic publication venues (e.g., [42]). While basement features as desired in the project are not part of the Vistas dataset or the Cityscapes dataset, the publications from Mapillary and the teams participating in the competitions are valuable resources.

### Task 2: Scene Reconstruction

Subsequent to detecting and delineating the objects of interest in Task 1, we need to infer the real world geometries of the objects. The task is known as scene reconstruction. A common scenario is that an object (e.g., a building or a basement feature) appears in multiple images. We can use all images of an object as long as the object is sufficiently visible in the image. Redundancy can help improve reliability of the detection. The scene reconstruction task helps automatically register different images of the same object so that we obtain information at the object level instead of the image level. Automatically reconstructing depth information and real world geometry of an object from a single, monocular image of the object is not impossible but is significantly challenging. Mertan et al. [43] provided a recent review on the topic. Available methods range from those that rely on hand-crafted features and rules (e.g., [44, 45]) to modern supervised ML (e.g., [46–48]) and unsupervised ML methods (e.g., [49, 50]).

When multiple images of a scene are available, the depth information can be estimated more easily and accurately by performing SfM [27] and Multi-view Stereo (MVS). SfM recovers the relative poses of the images while MVS generates a dense depth map for each image. While many Mapillary data were captured in sequence, Mapillary data are sub-optimal for SfM analysis due to the low overlapping ratio between images, complex motion trajectories, under-constrained camera parameters, and the presence of non-stationary objects (e.g., pedestrians, vehicles). Off-the-shelf SfM algorithms have limited success in reconstructing objects from Mapillary data. However, with appropriate calibration and tuning, the Mapillary Research team and collaborators had successfully demonstrated that scene reconstruction from Mapillary data is possible [51]. The research led to the publication of a set of 750,000 Mapillary images with depth information. Antequera et al. [51] also showed that such a dataset can be used to train single-image-depth networks to predict depth information from individual images. The methodology and data introduced in [51] will potentially be the basis for scene reconstruction in this project.

### Incorporating Quality Assessment as Part of the Data Analysis

The diversity in Mapillary data quality as described in Section 3.4 creates opportunities as well as challenges. Together with the large amount of data available, the data diversity provides potential for creating models that have high degrees of generalisability and robustness [52, 53]. A high level of diversity means that the dataset captures a wider variety of representations. ML models trained with more diverse data have a better capability to generalise, as they capture a variety of representations and are less prone to overfitting [54]. However, low-quality and defected images may impede the performance of computer vision models. There are two general directions for addressing image quality issues. In the first direction, images from the initial data pool are filtered by an image quality assessment before being inputted into the target analysis (e.g., Tasks 1 & 2 described in Sections 4.2–4.3). That will be a no-reference/blind image quality assessment [55] as higher quality references are not available. Many solutions are available for the task, including solutions based on DL, can be traced from review papers such as [56]. In the second direction, we can train an image selection neural network simultaneously with an image segmentation neural network (i.e., Task 1) in a reinforcement learning process (e.g., [57]). The former network learns to select/reject images through maximising the performance of the latter network in performing the target task. The second direction is more sophisticated, potentially more complex and does not require image quality labels. In addition to data quality issues, data incompleteness is a known issue that can impact the analysis output. For example, false negatives are unavoidable where suitable input data are missing. Those must be taken into account when interpreting results from Mapillary data.

## Preliminary Experiment

To demonstrate the data analysis approach presented in Section 4 and investigate the suitability of Mapillary images for the intended purpose, a simplified, small scale experiment was conducted. As a preliminary experiment, the investigation is restricted to Mapillary data captured around the Merrion Square area in Dublin city (see Fig. 1). Most buildings in the area are Georgian terraced houses which have front basements protected by railings visible from the street view. Basement railings are selected as the target object type for the image segmentation task in this experiment. The Merrion Square park in the area is also surrounded by ion railings which have no relation to basement structures. That mix of basement and non-basement railings creates an interesting setting for the experiment.

### Data Preparation

The sample dataset in Merrion Square consists of 217 images belonging to nineteen sequences. An image sequence in the Mapillary database is a series of consecutive images captured by a unique user with a unique equipment in one session. A manual, subjective assessment was performed for the sample dataset. Each image was assessed by a human annotator on the basis of whether the image is sufficiently clear to indicate the presence or absence of basement features. Notably, while many objective image quality metrics are available for quantifying blurriness, noise, and colour inaccuracies (e.g., [58]), subjective assessment is considered accurate and reliable. 157 images (i.e., 72.4% of the initial image set) were found sufficient for the target purpose. Among the images deemed unsuitable, 90.0% were blurry, mostly due to motion blur. Motion blur is common for images taken in low light conditions (e.g., near dawns and dusks) and often affects multiple images in the same sequence. The two other issues identified in the sample batch are out of focus (6.7%) and being in a corrupted file (3.3%). While the assessment is subjective and has too few samples to represent the overall characteristics of Mapillary data, the results suggest that Mapillary data have the potential to be useful for basement feature detection.

Subsequent to passing the quality assessment, the images were manually annotated by drawing polygonal regions around basement railings. We deliberately included the background behind the railings (e.g., building façades) as part of the segment annotations since the background is likely helpful in distinguishing basement and non-basement railings. Non-basement railings (e.g., those around the park) were not annotated and are considered as a part of the background. Among the 157 images, 115 images contain a basement railing feature and 42 images contain background only.

### Training and Evaluating Basement Railing Segmentation Models

We adopted the YOLOv5 instance segmentation algorithm^6^. YOLOv5 provides a number of models at different sizes. Larger models provide higher levels of accuracy while requiring more memory as well as time for training and inference. YOLOv5l, the largest model that fits within our 40GB GPU memory, was selected as accuracy is preferred over speed. The model was pre-trained with the COCO dataset [59]. We exploited the low level features learned from the COCO dataset in the pre-trained model by freezing the first nine layers of the model and trained only the remaining layers. That technique is known as transfer learning [60] and is effective when the amount of data available for training is small. The model was trained with stochastic gradient decent for 100 epochs with a batch size of 16 images. YOLOv5 automatically applies multiple data augmentation techniques including moisaic, cutout, and different geometric and colour transformations. The input image size for the model was set to 1280 pixels.

**Table 1:** Basement railing segmentation accuracy

	AP	Precision	Recall
Run 1	86.7%	84.4%	80.8%
Run 2	86.3%	83.1%	81.3%
Run 3	82.4%	85.8%	80.5%
Run 4	80.7%	78.3%	77.7%
Median	84.4%	83.8%	80.6%
Std. Deviation	2.9%	3.3%	1.6%

**Fig. 6:**
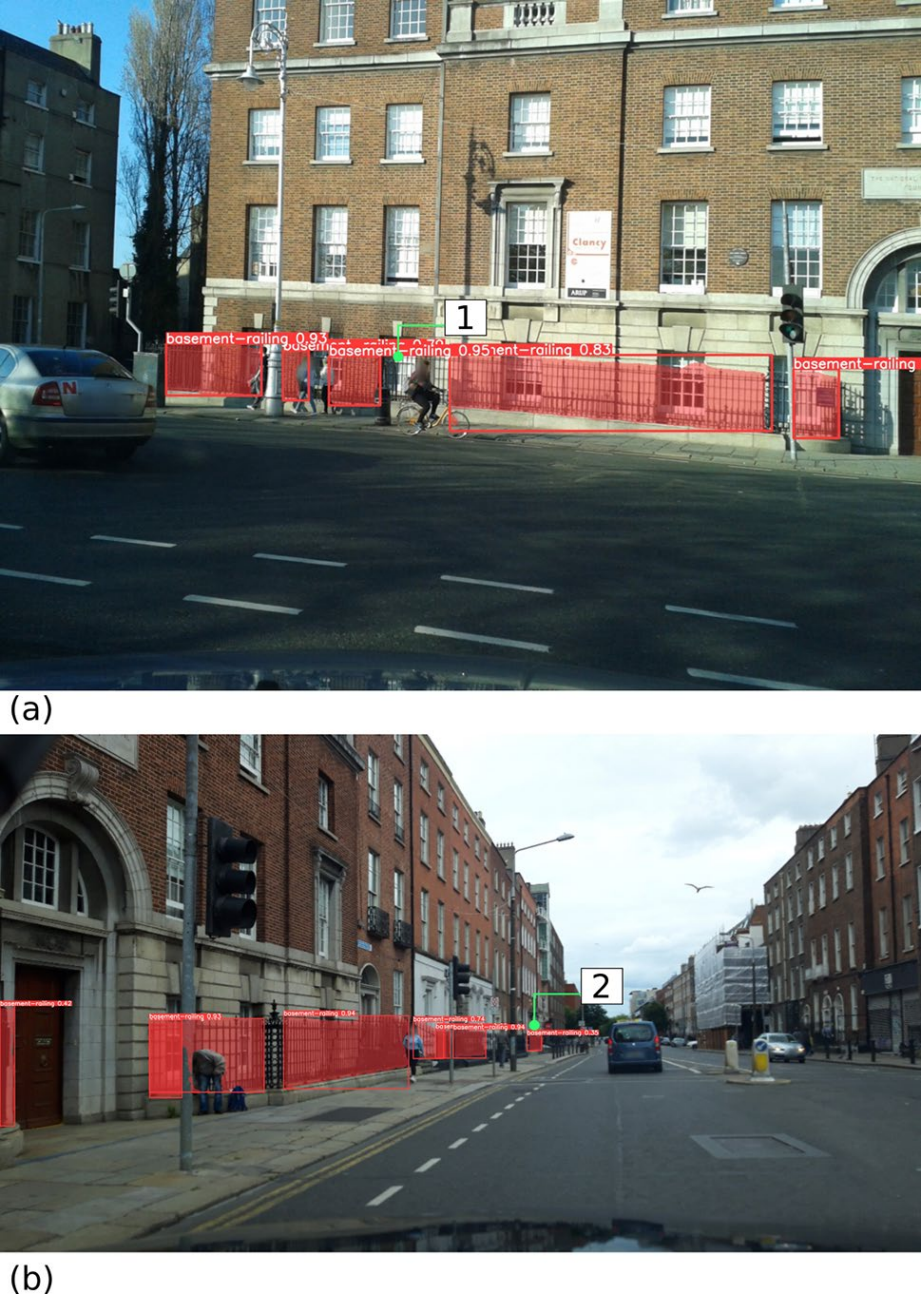
Examples of prediction results of basement railings

**Fig. 7:**
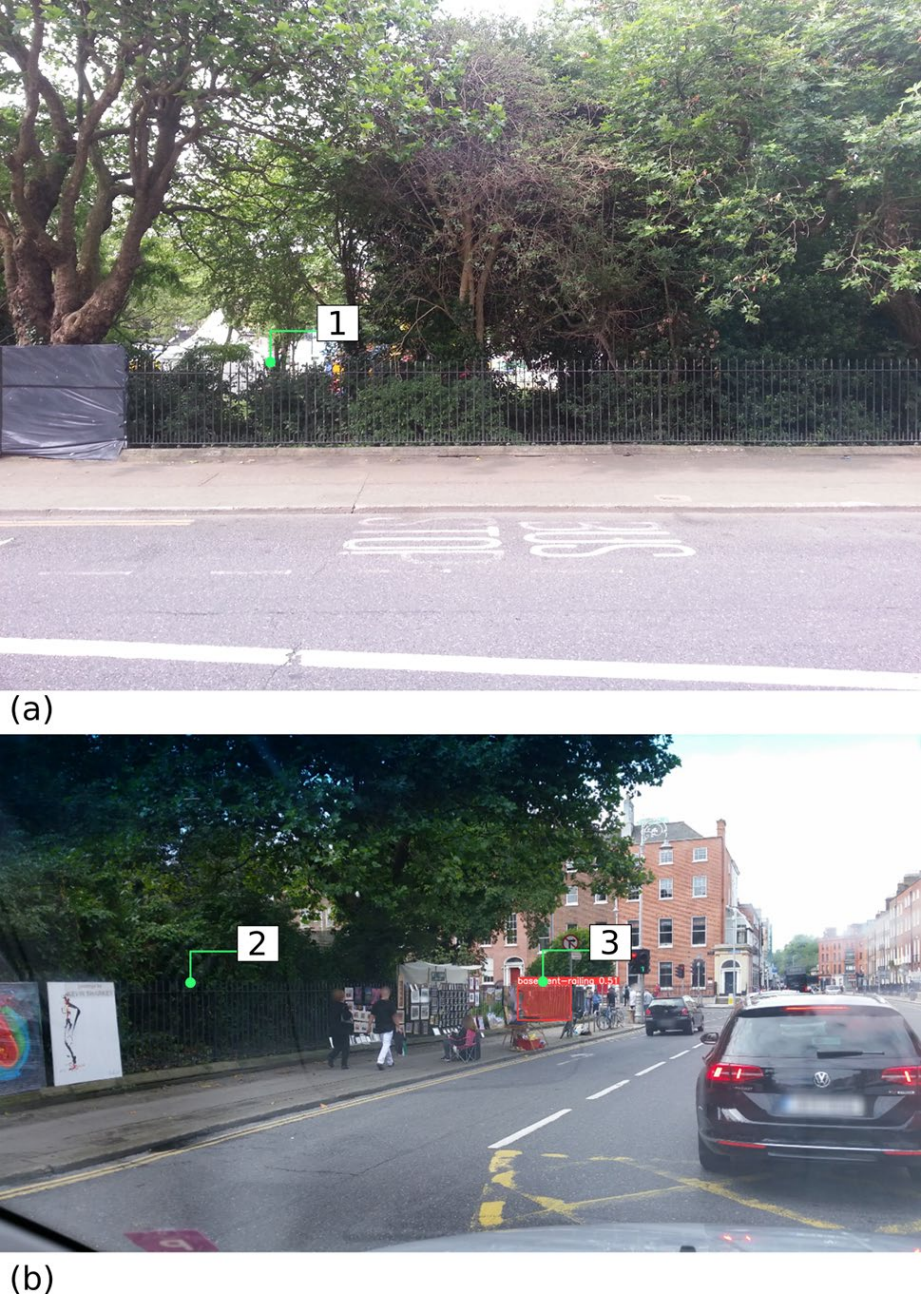
Examples of prediction results of non-basement railings

Since the number of sample images is small, the rigorous k-fold cross validation method with $k=4$ was selected. The image set was randomly partitioned into four parts, each part has from 39 to 40 images. The model was trained in four runs. Each run was trained using a different set of three data parts and evaluated against the remaining part. The evaluation results of the four runs are presented in Table 1. The precision, recall, and Average Precision (AP) scores in the table were calculated using an Intersection over Union (IOU) factor of 50%. The median precision, recall and AP scores of the four runs were all higher than 80%. Even though the experiment was conducted in a simplified context (i.e., single class, small dataset from a restricted geographical area with builing style uniformity), the high performance scores achieved in the experiment demonstrated the feasibility of detecting basement features from Mapillary imagery data.

After the k-fold evaluation, we trained a final model with all 157 images available. The performance of the final model is shown in Figs. 6 and 7. Notably, those images belong to Mapillary sequences never been exposed to the model during training. Namely, the images were captured by contributors and equipment not previously seen by the model. In Fig. 6, the model demonstrated a high level of accuracy in detecting basement railings. In one instance (i.e., location [1] in Fig. 6a), a small segment of railing behind a cyclist was missed. In another instance (i.e., location [2] in Fig. 6b), an unclear segment of railing appearing at a distance was successfully detected by the model. In most cases, the model correctly recognised non-basement railings (e.g., location [1] in Fig. 7a and location [2] in Fig. 7b). However, in a few instances (e.g., location 3 in Fig. 7b), the model mistook non-basement railings for basement railings. In the particular case of location 3 in Fig. 7b, the building behind the railing segment may confuse the model.

### Evaluation of Georeferencing Information

**Fig. 8:**
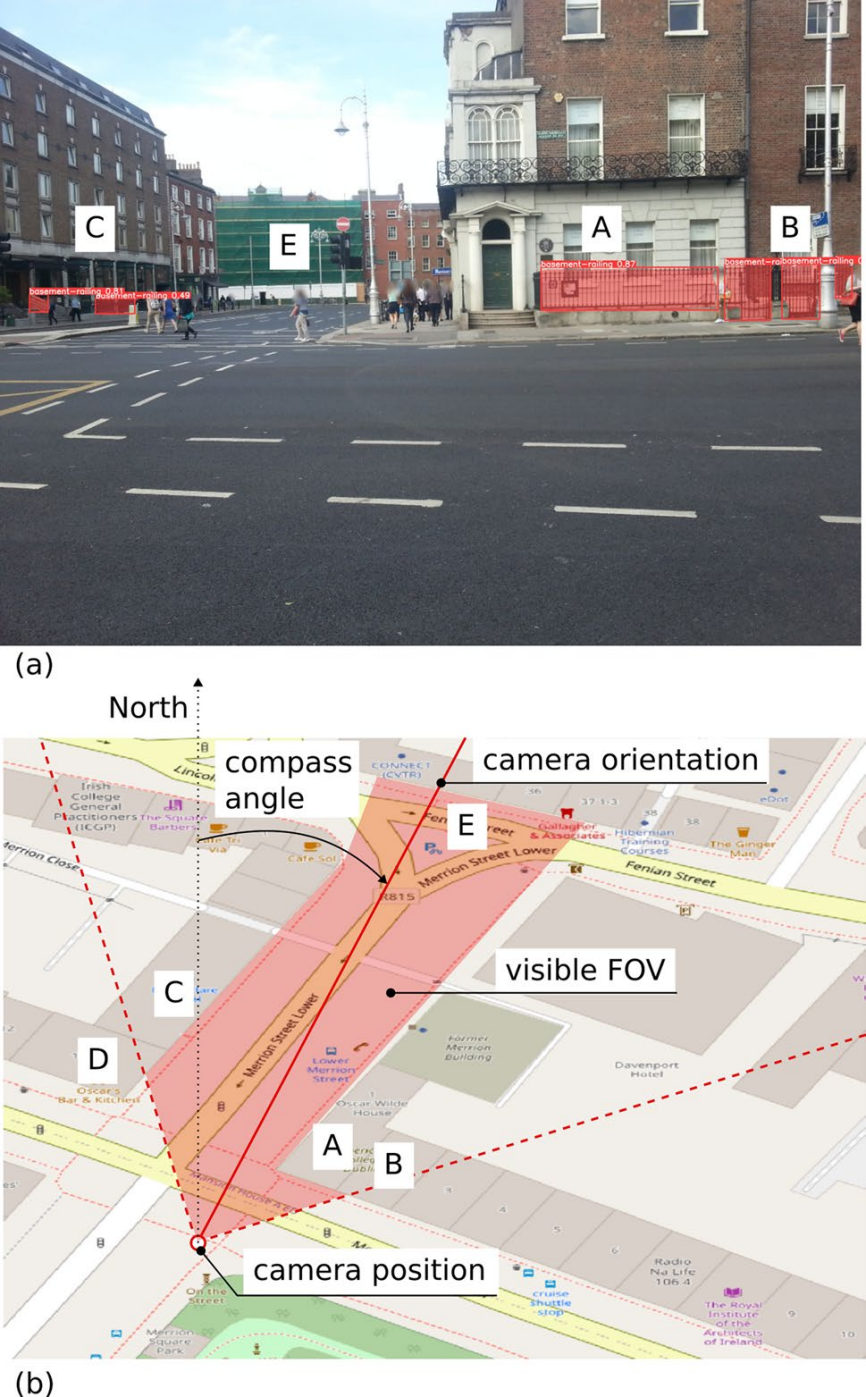
Identify buildings and detected features by manual georeferencing

To evaluate the metadata offered by Mapillary for geolocating objects in an image, we manually georeference one example image as shown in Fig. 8a. The API described in Section 3.2 allows retrieving the camera position (i.e., latitude and longitude) and orientation (i.e., compass angle) corresponding to that image. The exact script for retrieving the metadata is available in the Github reposition accompanying this paper (see Appendix A). The orientation and position of the camera were then plotted atop an Open Street Map basemap with buildings outlines (see Fig. 8b). The objects appearing in the FOV were then estimated by casting lines of sight from the camera position towards the directions on the left and right of the camera axis until the lines reach an obstruction such as a building. The shaded area in red colour in Fig. 8b is the estimated visible FOV.

While neither the Mapillary metadata or the building outlines in Open Street Map are perfectly accurate, the manual georeferencing did allow us to identify most buildings in Fig. 8a. Specifically, buildings denoted as A, B, C, and E in Fig. 8 were successfully identified through the line of sight casting. Building D does not appear in the image but was incorrectly located within the FOV in Fig. 8b. Nevertheless, the evidence obtained from the experiment suggests that the georeferencing information provided by Mapillary was useful for geolocating the objects in the image. Using the information, scene reconstruction such as that described in Section 4.3 can be carried out to automate the operation.

## Concluding Remarks

This paper describes an ongoing project on extracting basement information from SVI data to enhance flood risk assessments. The paper provides justification for why basement information is important to flood assessments and discusses basement features detectable from the street view. The features identified through data observations include basement railings, basement wells, skylights, ventilators, basement windows and doors. That list is not exhaustive and can be extended by consulting architecture archives. Two candidate SVI data sources, GSV and Mapillary, are available in the study area in Dublin. For the AOI in the project, GSV data have better spatial coverage and are of high quality. The data quality of Mapillary data is heterogeneous. There are many images suitable for the project but also images that suffer from insufficient quality, unsuitable FOV, file corruption, among various issues. Mapillary data have a more even temporal distribution and much richer image metadata. However, GSV’s Terms of Service do not allow bulk download, make an offline copy of GSV data, or create derived information products from the data. That makes Mapillary the only choice in the project.

The paper also discusses various considerations towards developing a data analysis strategy. The considerations include the formulation of the target tasks as a two step procedure consisting of image segmentation and scene reconstruction. Discussions on available algorithms and resources (e.g., data, papers) for each task are also provided. As image quality issues likely cause a significant challenge, the paper explains two potential directions for incorporating image quality assessment in the data analysis pipeline. Despite the significant challenges caused by the inherent nature of crowdsourced data, the tasks defined in the project can be achieved thanks to the wealth of open data, source codes, and closely related research. While many potential tools have been identified, the exact capabilities of the tools have not been evaluated.

Preliminary experiments were conducted to evaluate the suitability of Mapillary data for the target tasks and to demonstrate the proposed data analysis strategy. The first experiment showed that a DL model trained using Mapillary images could detect and segment basement railings at an average precision of over 80%. Interestingly, the model was able to distinguish basement versus non-basement railings in most cases. In the second experiment, the image metadata provided by Mapillary was successfully used to geolocate buildings captured in an image. The evidence available from the preliminary experiments confirms the suitability of Mapillary data and the feasibility of the presented approach in a simplified context (i.e., homogeneous architectural styles in a limited geographical region). The robustness of the solution in more complex, real-life scenarios will be investigated in future research.

Issues related to data quality remain a challenge to be addressed. For the preliminary experiment, data are manually filtered based on the annotator’s subjective judgement of whether the images show the presence or absence of basement features. Another limitation of the proposed approach is that information obtainable from the street view may be incomplete. For example, not all buildings that have a basement have relevant features viewable from the public right of way. In addition, important information such as basement levels and volumes are less discernible from street-view images. While SVI can provide useful information, its combination with other potentially available dataset (e.g., aerial images, laser scanning data, GPR data, building registries) would create a more complete knowledge of basement structures.

Nevertheless, compared to methods relying on laser scanning or GPR data, the method proposed in this paper can be more cost-effective and scalable. Street view imagery data are widely available at a low or no cost. When necessary, new data captures are relatively easy such as by setting up consumer-grade cameras and positioning equipment aboard a wide variety of vehicles such as buses, police cars, or volunteer private cars. Valuable information about basement structures can be extracted from the available data using the wealth of free and open-source computer vision algorithms and tools. In addition, as SVI data are ubiquitous in many urban areas, SVI datasets capture a large range of basement indicators for different building types. Due to the significantly higher availability of SVI data, analyses based on SVI data are expected to be more robust compared to those relying on LiDAR or GPR data. We acknowledge that the research is in its early stages, and the methodology may need further refinement in light of evidence appearing from more systematic evaluations.

## Appendix A Coding scripts

The scripts for downloading Mapillary data and for training the basement railing segmentation model are available at https://github.com/av-vo/basement-railing-segmentation.git.

## Footnotes

1 https://www.floodinfo.ie/map/past_flood_event_user_guidance_notes/.

2 https://www.floodinfo.ie/map/pf_addinfo_report/11703/.

3 https://www.google.com/intl/en-GB_ALL/permissions/geoguidelines/.

4 https://github.com/ultralytics/yolov5.

5 https://www.mapillary.com/dataset/vistas.

6 https://github.com/ultralytics/yolov5.
